# Computational modelling of the cerebral cortical microvasculature: effect of x-ray microbeams versus broad beam irradiation

**DOI:** 10.1088/1361-6560/aa68d5

**Published:** 2017-04-13

**Authors:** A Merrem, S Bartzsch, J Laissue, U Oelfke

**Affiliations:** 1 Biomedizinische NMR Forschungs GmbH am Max-Planck-Institut für biophysikalische Chemie, Am Fassberg 11, 37077 Göttingen, Germany; 2 Klinikum Rechts der Isar, Ismaninger Str. 2, 81675 München, Germany; 3 The Institute of Cancer Research, Royal Marsden Hospital, Fulham Rd, London SW3 6JJ, United Kingdom; 4 University of Bern, Hochschulstrasse 4, 3012 Bern, Switzerland; 5 This work was carried out at the German Cancer Research Center, Im Neuenheimer Feld 242, 69120 Heidelberg, Germany; amerrem@gwdg.de

**Keywords:** blood vessels, microbeam radiation therapy, computer simulation, vascular trees, synchrotron radiation, radiation damage, mathematical modeling

## Abstract

Microbeam Radiation Therapy is an innovative pre-clinical strategy which uses arrays of parallel, tens of micrometres wide kilo-voltage photon beams to treat tumours. These x-ray beams are typically generated on a synchrotron source. It was shown that these beam geometries allow exceptional normal tissue sparing from radiation damage while still being effective in tumour ablation. A final biological explanation for this enhanced therapeutic ratio has still not been found, some experimental data support an important role of the vasculature. In this work, the effect of microbeams on a normal microvascular network of the cerebral cortex was assessed in computer simulations and compared to the effect of homogeneous, seamless exposures at equal energy absorption. The anatomy of a cerebral microvascular network and the inflicted radiation damage were simulated to closely mimic experimental data using a novel probabilistic model of radiation damage to blood vessels. It was found that the spatial dose fractionation by microbeam arrays significantly decreased the vascular damage. The higher the peak-to-valley dose ratio, the more pronounced the sparing effect. Simulations of the radiation damage as a function of morphological parameters of the vascular network demonstrated that the distribution of blood vessel radii is a key parameter determining both the overall radiation damage of the vasculature and the dose-dependent differential effect of microbeam irradiation.

## 1. Introduction

Microbeam radiation therapy (MRT) is an emerging pre-clinical treatment strategy for cancer and considered as a promising alternative to ablate brain tumours in children (Laissue *et al* 2007, Slatkin *et al* 2009). In MRT, a filtered white beam of synchrotron x-ray radiation with a typical energy spectrum of 50–600 keV (Crosbie *et al* 2015) passes through a collimator with equidistant, 10–100 *µ*m wide slits separated by an inter-slit distance of a few hundred microns before reaching the patient. This leads to the exposure of equidistant, quasiparallel tissue slices with a high peak dose in the order of hundreds of Gray delivered at very high dose rates while the tissue in between receives far lower radiation doses. Microbeam dose distributions typically have a minimum, i.e. valley dose of 5–20 Gy (for review see Bräuer-Krisch *et al* (2010)). In pre-clinical studies with rats and mice, it was demonstrated that MRT selectively damages tumorous tissues (Laissue *et al* 1998, Dilmanian *et al* 2002, Bouchet *et al* 2010, Crosbie *et al* 2010) and spares normal tissue better from radiation damage than broad beam irradiation at a comparable tumour control rate (Dilmanian *et al* 2003a, Bouchet *et al* 2016).

The biological processes responsible for this effect are not yet understood. Recent work suggests that the enhanced sparing of normal tissue is associated with a differential sensitivity of vasculature between tumour and normal tissues. Bouchet *et al* (2013) observed that following microbeam irradiation (MBI) of brain tumor bearing rats, the oxygen supply in neoplastic brain tissue was impaired due to vascular radiation damage whereas oxygen supply in normal tissue remained unaffected at equal radiation doses. In this current study, we compared the effects of broad-beam irradiation (BB) and MBI on a normal vascular network using computer simulations to better understand the sparing effect of MRT on normal tissue. Furthermore, we examined the influence of beam spacing, beam direction, the ratio of peak to valley dose (PVDR) and vascular network morphology on the damage inflicted on the vasculature. For our simulations we developed two computational models: an anatomical model of the microvasculature of the cerebral cortex and a radiation damage model which yields a dose-dependent probability of a blood vessel to remain functional (‘survival probability’).

In accordance with previous findings on the morphology of the cerebral microvasculature (Duvernoy *et al* 1981, Lorthois and Cassot 2010), the anatomical model consists of two components: a series of vascular trees (i.e. a vascular forest), representing the arterioles and venules, and a mesh-like capillary system. For the algorithmic generation of vascular trees, a variety of methods have been published (Schwen and Preusser 2012). Our algorithm is based on the principle of constrained constructive optimization (CCO), i.e. the construction of a vasculature that optimally supplies a given tissue volume under constraints determined by *a priori* knowledge of vascular geometry and the rheology of blood (Schreiner and Buxbaum 1993). This concept has been extensively used for simulating vascular trees (e.g. Karch *et al* (1999), Schreiner (2001), Kretowski *et al* (2003) and Hamarneh and Jassi (2010)). We chose a CCO-based approach for the modelling of arterioles and venules to adapt the vasculature to known anatomical and physiological parameters. We then modelled the capillary mesh as a Voronoi diagram in three dimensions, thereby extending a previous 2D model (Lorthois and Cassot 2010). All blood vessels are modelled as rigid cylinders connecting the nodes of the network.

Our radiation damage model is based on a known dose-dependent survival probability of endothelial cells in the blood vessel wall and on the assumption that a blood vessel will become dysfunctional if it develops a sufficiently large leak in the endothelium. The model yields a ‘survival probability’ for each blood vessel depending on its length and radius. Using this model, we simulated radiation damage to the entire vascular network by deciding with a non-deterministic algorithm which blood vessels will become dysfunctional and removing those vessels from the vasculature. Radiation damage was simulated for (1) a homogeneous dose and (2) an idealized microbeam dose distribution at an equal integrated radiation dose (Dilmanian *et al* 2003b, Zhong *et al* 2003, Serduc *et al* 2014). The damage was evaluated by computing the total length of the vascular network remaining after irradiation and the distribution of distances between geometric points spread homogeneously throughout the tissue and the nearest functional blood vessel (tissue-vessel distances). We also computed the effects of vascular morphological changes on the inflicted radiation damage of the vasculature using a reduced anatomical model without capillaries.

## 2. Methods

### 2.1. Anatomical model

**Figure 1.**
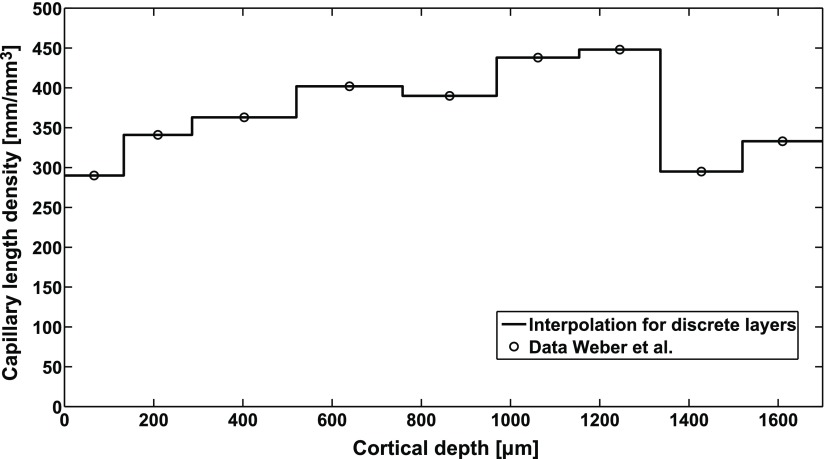
Dependence of the capillary length density *d*(*z*) on the cortical depth. The circles represent published data (Weber *et al* 2008, figure 4(A)), the line is an interpolation based on the assumption that the length density changes discretely between cortical layers.

Our anatomical model is based on the macaque model described by Weber *et al* (2008). The model represents a cuboid of cortical tissue with a length and width of 2.5 mm and a cortical depth of 1.7 mm. This allows for simulations on a spatial scale which is realistic for the clinical MRT scenario of treating brain tumours in children (Laissue *et al* 2007, Slatkin *et al* 2009). Moreover, the availability of depth-dependent quantitative data on vascular parameters for the macaque model (Weber *et al* 2008, figure 4(A)) enables a simulation of the layered structure (Duvernoy *et al* 1981, Hirsch *et al* 2012) of the cerebral cortex. In the anatomical model, the vascular forest of arterioles and venules supplies and drains the cortical tissue volume from a set of root nodes at the surface. The terminal nodes of the vascular trees are connected with the capillary network which has a 3D mesh-like structure with a layer-dependent mesh size. The modelled microvascular geometry is fitted to the experimental parameters displayed in table 1 and figure 1 to simulate anatomically plausible blood vessel radii, arterial and venous branching complexity, and capillary mesh sizes.

**Table 1.** Anatomical and physiological parameters used for modelling the cortical vasculature.

Parameter	Symbol	Value
Area density of arterial root points (Weber *et al* 2008)	${{n}_{\text{A}}}$	$3.85\,\text{m}{{\text{m}}^{-2}}$
Area density of venous root points (Weber *et al* 2008)	${{n}_{\text{V}}}$	$2.44\,\text{m}{{\text{m}}^{-2}}$
Mean perfusion in the cerebral cortex (Kretschmann *et al* 1986)	$\langle Q\rangle $	$78.75\ast {{10}^{-2}}\,{{\min}^{-1}}$
Radius of arterioles at the cortical surface (Duvernoy *et al* 1981)	${{r}_{\text{A}}}$	$17.5\,\mu \text{m}$
Radius of venules at the cortical surface (Duvernoy *et al* 1981)	${{r}_{\text{V}}}$	$32.5\,\mu \text{m}$
Radius of arterioles and venules at terminal nodes (Weber *et al* 2008)	${{r}_{\text{T}}}$	$4\,\mu \text{m}$
Mean radius of arterioles and venules (Weber *et al* 2008)	$\langle r\rangle $	$5.07\,\mu \text{m}$
Mean density of arterial branching nodes (Cassot *et al* 2009)	${{c}_{\text{A}}}$	$155\,\text{m}{{\text{m}}^{-3}}$
Mean density of venous branching nodes (Cassot *et al* 2009)	${{c}_{\text{V}}}$	$114\,\text{m}{{\text{m}}^{-3}}$
Blood viscosity (Holsworth and Wright 2012)	$\eta $	$4\,\text{mPa}\,\text{s}$
Mean capillary radius (Cassot *et al* 2006)	${{\langle r\rangle}_{\text{cap}}}$	$2.955\,\mu \text{m}$
Standard deviation of capillary radius (Cassot *et al* 2006)	$ \Delta {{r}_{\text{cap}}}$	$0.65\,~\mu \text{m}$
Length density of capillary network (Weber *et al* 2008)	$d(z)$	See figure 1

#### 2.1.1. Arterioles and venules.

The vascular forest is constructed to fit the parameters shown in table 1. Additionally, we define a depth-dependent perfusion $Q(z)$, where the cortical depth z is the distance from the cortical surface. $Q(z)$ is proportional to the metabolic demand of the different cortical layers as measured with COX staining in a Macaque (figure 4(A) in Weber *et al* (2008)) and normalized to the mean perfusion value shown in table 1. Besides these experimental parameters, the construction of the vascular forest is determined by the following constraints: First, the pressure difference between all root nodes and all terminal nodes of one kind (arterial or venous) shall be the same, these differences are denoted by ${{P}_{\text{A}}}$ and ${{P}_{\text{V}}}$. Second, Hagen–Poiseuille’s law (Vogel 1994) shall be valid for all blood vessels. This physical law describes the relation between the flow and the pressure gradient in a tubular structure under the assumption of laminar flow. Third, the scaling of blood vessel radii at branching nodes shall obey Murray’s law: $r_{0}^{\gamma}=r_{1}^{\gamma}+r_{2}^{\gamma}\,$ with vessel 0 branching into vessels 1 and 2 and the Murray exponent $\gamma &gt;0$.

We achieved the construction of vascular forests with the described input parameters and constraints with a two-level optimization algorithm consisting of an inner and an outer optimization. In the inner optimization, a vascular forest is generated using a set of ‘tree-building parameters’. These are partly taken from literature: the area densities of arterial and venous root nodes ${{n}_{\text{A}}}$ and ${{n}_{\text{V}}}$, the mean perfusion $Q$, and the blood viscosity $\eta $. The remaining tree-building parameters are ${{P}_{\text{A}}}$ and ${{P}_{\text{V}}}$, the mean point densities of arterial and venous terminal nodes ${{\rho}_{\text{A,V}}}$, and the Murray exponents ${{\gamma}_{\text{A,V}}}$, whose values are computed in the outer optimization.

**Figure 2.**
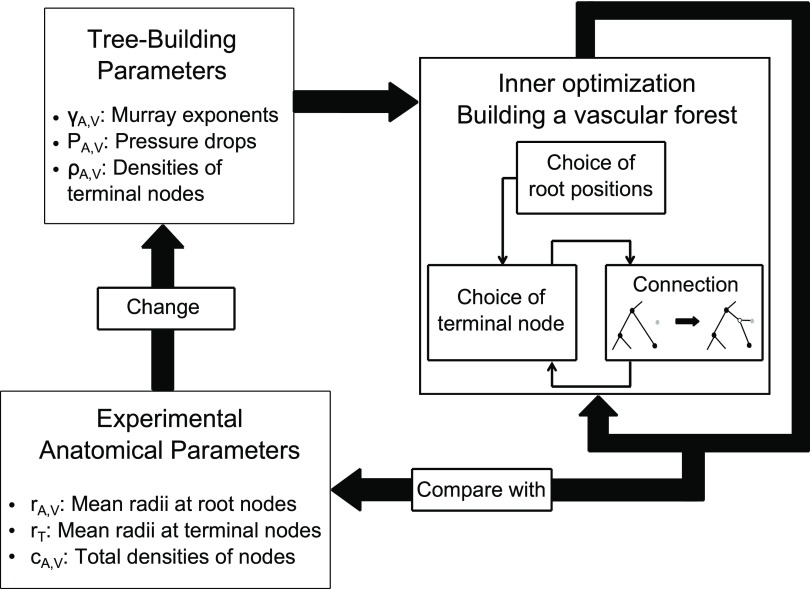
Overview of the anatomical modelling algorithm for arterioles and venules. A vascular forest is built to minimize the blood volume under constraints which are defined by the tree-building parameters. Six of the tree-building parameters must be adjusted for the vascular forest to fit the experimental anatomical parameters. This is done by iteratively minimizing cost functions which are used to compare the simulated vasculature to the reference parameters.

The first step of the inner optimization is the choice of ${{R}_{\text{A}}}=24~$ arterial and ${{R}_{\text{V}}}=15~$ venous root node positions on the cortical surface to achieve the area densities ${{n}_{\text{A,} ~\text{V}}}$ as shown in table 1. The points are chosen randomly but with an appropriate minimum distance to root points of the same type to ensure a homogeneous distribution. Then, terminal nodes are iteratively added to the vasculature. Their positions are randomly chosen having minimum distances to other terminal nodes and a point density proportional to $Q(z)$ with a mean of ${{\rho}_{\text{A}}}$ or ${{\rho}_{\text{V}}}$, respectively. Each new terminal node is connected to the vascular tree originating from the nearest root node using the method developed by Hamarneh and Jassi (2010) for their CCO-based algorithm VascuSynth which generates vascular trees. This adds a new branching to the vascular tree as visualized in figure 2. The position of the new branching node is chosen to minimize the total volume occupied by the blood vessels under the constraints of Hagen–Poisseuille’s and Murray’s laws, with a blood flow of $\langle Q\rangle \rho _{\text{A}}^{-1}$ for arterial nodes or $\langle Q\rangle \rho _{\text{V}}^{-1}$ for venous nodes. Omitting the constraint that each terminal node needs to be connected to the nearest root node leads to large random variations in the distribution of terminal nodes among the supplying root nodes. An example of vascular forests was generated with this modification to study the effect of vascular tree morphology on radiation damage.

**Figure 3.**
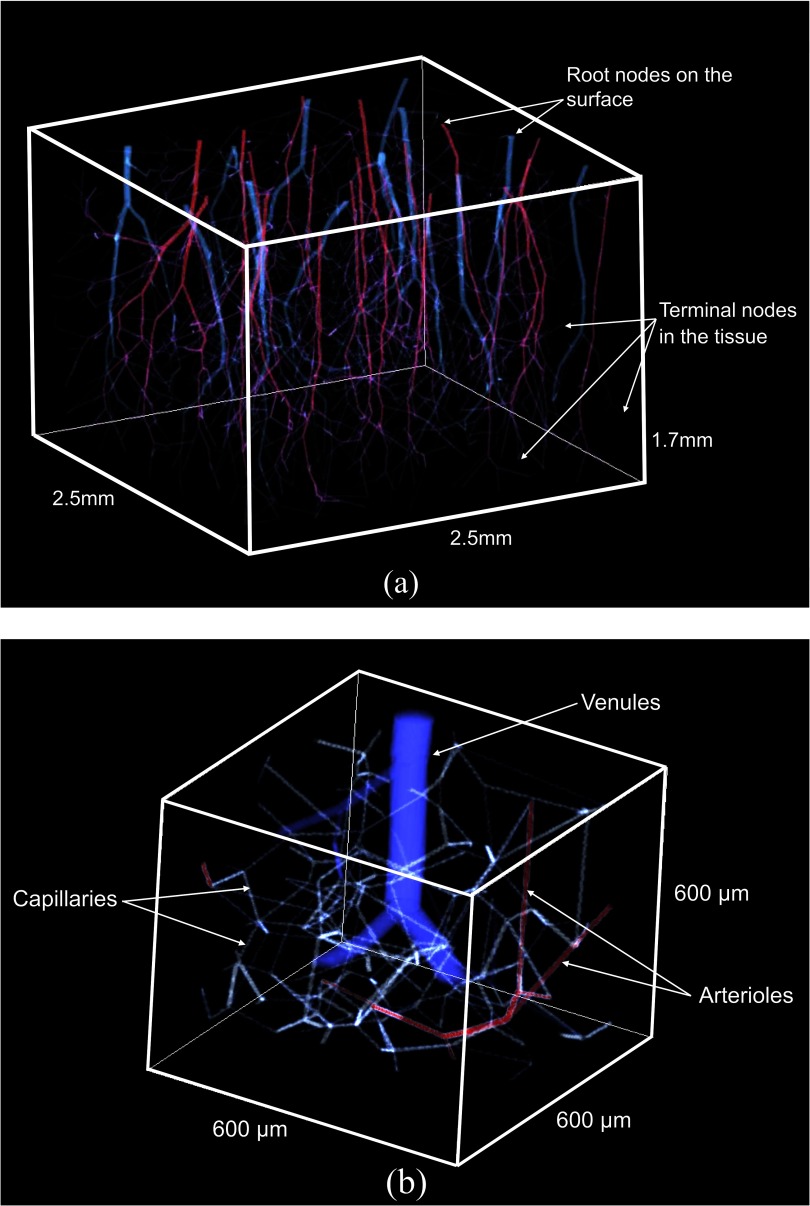
Visualization of the simulated vasculature. (a) Entire simulation domain with a typical vascular forest, arterioles in red, venules in blue. Blood from the surface supplies the simulated tissue volume. The total depth of the cerebral cortex of 1.7 mm corresponds to measurements in a macaque (Weber *et al* 2008). (b) Subset of the simulation domain with capillaries shown in white.

In the outer optimization displayed in figure 3, we adjusted the tree-building parameters ${{P}_{\text{A}}}$, ${{P}_{\text{V}}}$, ${{\rho}_{\text{A}}}$, ${{\rho}_{\text{V}}}$, ${{\gamma}_{\text{A}}}$, and ${{\gamma}_{\text{V}}}$ to fit the following anatomical parameters: the mean radii at arterial and venous root nodes ${{r}_{\text{A}}}$ and ${{r}_{\text{V}}}$, the mean radius at terminal nodes ${{r}_{\text{T}}}$, and the densities of arterial and venous branching nodes ${{c}_{\text{A}}}$ and ${{c}_{\text{V}}}$. For each of the six tree building parameters, we constructed a cost function $F\left({{r}_{\text{A}}},{{r}_{\text{V}}},{{r}_{\text{T}}},{{c}_{\text{A}}},~{{c}_{\text{V}}}\right)$ (see table 2) measuring those deviations of the simulated anatomical parameters from the reference values which are determined by the respective tree-building parameter. Each cost function was minimized by evaluating its mean and standard deviation on vascular forests generated with varying values of the respective tree-building parameter and applying a quadratic fit in a 3*σ* neighbourhood of the lowest sampled value.

**Table 2.** Tree-building parameters and cost functions for adapting the simulated vasculature to the anatomical parameters. The subscript ‘sim’ refers to simulated values, the subscript ‘lit’ refers to values given in the literature, (table 1). The subscripts ‘*A*’ and ‘*V*’ symbolize arterioles and venules.

Tree-building parameter	Symbol	Cost function
Densities of terminal nodes	${{\rho}_{\text{A,V}}}$	${{\left(\frac{{{c}_{\text{A,V;sim}}}}{{{c}_{\text{A,V;lit}}}}-1\right)}^{2}}$
Murray exponents	${{\gamma}_{\text{A,V}}}$	${{\left(\frac{{{\left(\frac{{{r}_{\text{A,V}}}}{{{r}_{\text{T}}}}\right)}_{\text{sim}}}}{{{\left(\frac{{{r}_{\text{A,V}}}}{{{r}_{\text{T}}}}\right)}_{\text{lit}}}}-1\right)}^{2}}$
Pressure drops	${{P}_{\text{A,V}}}$	${{\left(\frac{{{r}_{\text{A,V;sim}}}}{{{r}_{\text{A,V;lit}}}}-1\right)}^{2}}+{{\left(\frac{{{r}_{\text{T;sim}}}}{{{r}_{\text{T;lit}}}}-1\right)}^{2}}$

After generating a vascular forest with optimized tree-building parameters, we scaled all radii of the simulated arterioles and venules to a mean radius of 5.07 *µ*m to achieve consistency with the macaque model (Weber *et al* 2008). The algorithm for simulating arterioles and venules was implemented in C++ and contains source code from an open source implementation of VascuSynth. Figure 2 gives an overview of the algorithm for constructing a vascular forest, figure 3(a) shows a typical vascular forest of arterioles and venules generated with our algorithm.

#### 2.1.2. Capillaries.

The brain microenvironment is mainly regulated by the endothelium of capillaries and the short diffusion distances between neurons and capillaries. Therefore, the capillary network is essential when simulating vascular radiation damage. To generate a 3D capillary network, we first divide the simulation volume into a number of cubes *n* each with an edge length *l*. Then one point *p*_*i*_ is chosen randomly within each cube. The points *p*_*i*_ are used as seed points to construct a Voronoi diagram (Voronoi 1908, Lorthois and Cassot 2010), i.e. the simulation volume $V\subset {{\mathbb{R}}^{3}}~$ is divided into cells $~{{C}_{i}}=\left\{x\in V:~\|x-{{p}_{i}}\|&lt;\|x-{{p}_{j}}\|\forall j\ne i\right\}$ with $i,j=1,2,\ldots,n$. $\|\centerdot \|$ denotes the Euclidean distance. The edges where the surfaces separating two neighbouring cells intersect form a mesh-like structure which is our model capillary network.

To account for the layered structure of the cerebral cortex, we adapted the capillary network to experimental data on the depth-dependent capillary length density, i.e. length per unit volume $d(z)$, see figure 1. The length density in a simulated capillary network is determined by the cube edge length *l*: on a set of capillary networks generated for testing purposes, we observed $d=\alpha {{l}^{-2}}$ with $\alpha ~=5.75$. Hence, a cube edge length distribution $~l(z)=\sqrt{\alpha /d(z)}$ was chosen to represent the experimentally observed distribution $d(z)$. Since $l$ is on the same length scale as the distances between the data points of $d(z)$, the cubes containing the Voronoi diagram seed points were constructed with a length equal to the average value of $l(z)$ inside the cube.

The mean and standard deviation of the capillary radii, 2.955 *µ*m and 0.65 *µ*m, were taken from a study on human microvascular morphology by Cassot *et al* (2006); they do not differ substantially from the mean capillary radius of 2.715 *µ*m in the macaque model (see Weber *et al* (2008), table 1). In our model, each capillary segment is randomly assigned a radius from a Gaussian probability distribution with these parameters.

To connect the capillary network with the arterial and venous trees, each terminal node of the vascular forest replaces the nearest capillary node. All capillary nodes previously connected with this capillary node are connected to the terminal arterial or venous node. Additionally, all capillaries which are found to intersect with the tree-like part of the blood vessel system are removed. The algorithm for generating capillaries was implemented in Matlab, version R2012b, The MathWorks, Inc. A subset of a simulated capillary network is shown in figure 3(b).

### 2.2. Radiation damage model

We developed a radiation damage model for blood vessels based on data of single endothelial cell survival in mouse brains. The dose response model described by Lyubimova *et al* (2001, equation (1)) yields the following survival probability $v(D)$ for endothelial cells in the brains of untreated mice:

1 \begin{eqnarray*}v(D)=~0.77+\frac{0.23}{{{\left(1+\frac{D}{6.3~\,\text{Gy}}\right)}^{0.8}}}\end{eqnarray*}

Additionally, our model is based on the following assumptions: (i) Each blood vessel’s inner surface is modelled as a cylindrical surface consisting of endothelial cells. (ii) All endothelial cells have an area A, identical shapes and are ordered in a periodic pattern on the inner surface of the blood vessel. (iii) The endothelial cells respond to irradiation independently from each other. (iv) A blood vessel has two possible states: intact or destroyed. (v) Destroying a blood vessel requires the presence of a hole in the blood vessel wall, of any given shape, with at least an area of $F=\lambda \pi {{R}^{2}}$. λ is a model parameter which scales with the fraction of blood flow leaving the vessel, $R$ is the radius of the vessel lumen. Biologically, the lesion can be interpreted as either a leak in the vessel wall (in smaller vessels, especially capillaries) (Dimitrievich *et al* 1984) or as an area with denuded subendothelium, a lesion that leads to the formation of an occluding thrombus in a capillary or a larger blood vessel (O’Connor and Mayberg 2000). With these assumptions, the resulting blood vessel survival probability $P$ depends on the length $L$ and the radius $R$ of the vessel and the dose distribution $D(l)$ along the vessel and is described by

2 \begin{eqnarray*}P=\exp \,\left\{\frac{2\pi R}{A}{\int}_{0}^{L}\ln \left(1-{{\left[1-v\left(D(l)\right)\right]}^{\frac{\lambda \pi {{r}^{2}}}{A}}}\right)\text{d}l\right\}\end{eqnarray*}

To derive equation (2), we first consider the probability ${{P}_{\text{T}}}$ of an irradiation dose *D* destroying a specific pre-defined target area *F* containing $S=\lambda \pi {{r}^{2}}{{A}^{-1}}$ endothelial cells: ${{P}_{\text{T}}}={{\left(1-v(D)\right)}^{S\,}}$. One of the $S$ endothelial cells shall be defined as the primary cell of *F*. In a blood vessel segment with a length dl consisting of $N=$ $2\pi R{{A}^{-1}}\text{d}l$ endothelial cells, any of these *N* cells can be a primary cell of a target area congruent to F, assuming periodic boundary conditions. Hence, the survival probability of the section is equal to the probability of destroying none of the *N* possible target areas:

3 \begin{eqnarray*}P\left(\text{d}l\right)={{\left(1-{{P}_{\text{T}}}\right)}^{N}}={{\left(1-{{\left[1-v(D)\right]}^{S}}\right)}^{N}}={{\left(1-{{\left[1-v(D)\right]}^{\frac{\lambda \pi {{r}^{2}}}{A}}}\right)}^{\frac{2\pi R\text{d}l}{A}}}\end{eqnarray*}

For a blood vessel to be destroyed, a target area must be destroyed anywhere in the vessel. Thus, the survival probability of the entire blood vessel is the product of the survival probabilities of the sections. The integration of $\log \left(P\left(\text{d}l\right)\right)$ over $\text{d}l$ yields equation (2).

**Figure 4.**
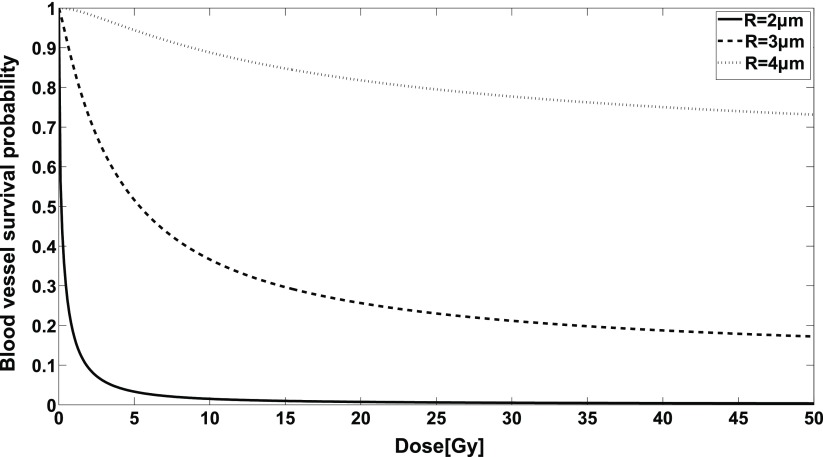
Radiation damage inflicted on blood vessels with typical simulated radii and a length of 50 *µ*m, computed with equation (2).

The model contains two parameters, $A$ and $\lambda $. We determined these parameters by fitting the model to experimental data on damage to a capillary network from irradiation measured by Dimitrievich *et al* (1984). The modelled radiation damage inflicted by homogeneous radiation doses is shown in figure 4 for typical microvessel radii.

### 2.3. Simulation and evaluation of radiation effects

#### 2.3.1. Simulated dose distributions.

**Figure 5.**
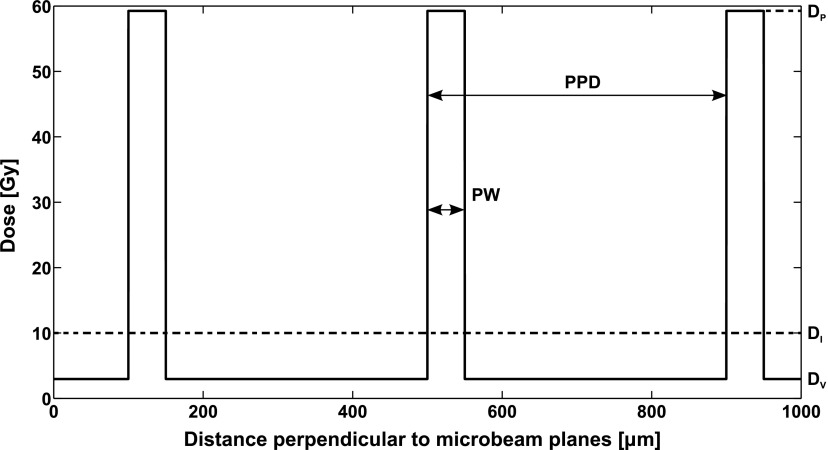
A cross section of the dose distribution for an array of ideal microbeams with an integrated dose of ${{D}_{\text{I}}}=10\,\text{Gy}$. Parallel tissue slices with a width $\text{PW}$ and a distance $\text{PPD}$ are irradiated unidirectionally with the peak dose ${{D}_{\text{P}}}$, the tissue in between receives a valley dose ${{D}_{\text{V}}}$. The peak-to-valley dose ratio $\text{PVDR}~={{D}_{\text{P}}}D_{\text{V}}^{-1}$.

To simulate radiation damage to the vasculature, we used two different dose distributions: one for the dose delivered by ideal microbeams, see figure 5, the other for a homogeneous dose delivered seamlessly by a broad beam (BB). To mimic experimental conditions (Dilmanian *et al* 2002, Serduc *et al* 2009, Bräuer-Krisch *et al* 2010), our ideal microbeams have a peak-to-valley dose ratio $\text{PVDR}~=~20$, a peak-to-peak distance $\text{PPD} ~ =~400\,\mu \text{m}$, and a peak width $\text{PW}=~50\,\mu \text{m}$. We used three different angles of the microbeam planes to the cortex surface, 0°, 45° and 90°; our results represent averages over all three incident angles. To allow a comparison with homogeneous irradiation at equal energy absorption, we characterize a microbeam dose distribution with its integrated dose ${{D}_{\text{I}}}=\left[{{D}_{\text{P}}}\text{PW}+{{D}_{\text{V}}}\left(\text{PPD}-\text{PW}\right)\right]~\text{PP}{{\text{D}}^{-1}}$. Radiation damage was simulated with integrated doses from 0 to 40 Gy. This corresponds to valley doses of up to 11.85 Gy and peak doses of up to 237.04 Gy.

#### 2.3.2. Simulations of radiation damage.

The first step of our simulation algorithm for radiation damage is to superimpose an idealized BB or MBI dose distribution with the modelled vascular geometry and assign a survival probability $P$ to each blood vessel according to equation (2). A random number $X$ between 0 and 1 is generated for each blood vessel, and if $X~&gt;~P$, the blood vessel is classified as destroyed and discarded from the modelled vasculature. Additionally, all blood vessels which no longer belong to an intact path between an arterial and a venous root point are discounted. Using this method, we simulated irradiation of the modelled vascular network with MBI and BB distributions and evaluated the vascular damage at equal total energy absorption.

#### 2.3.3. Effect of vascular tree morphology.

In addition to the simulations on the entire vascular network, we investigated the effect of morphological asymmetries in the vascular forest on its sensitivity to radiation damage. In a vascular forest built with the constraint that all terminal nodes must be connected to the nearest root node, all root nodes will contribute to the blood supply or drainage (example shown in figure 3(a)). In vascular forests built without this constraint (unconstrained forests), the distribution of terminal nodes among the vascular trees is determined by the order in which terminal node positions are chosen. This leads to large and randomly varying asymmetries in this distribution. We characterized these asymmetries by the normalized Shannon Entropy $H=-\ln {{(N)}^{-1}}{\sum}_{i=1}^{N}{{P}_{i}}\ln \left({{P}_{i}}\right)$ (Shannon and Weaver 1963). $N$ is the number of either arterial or venous root nodes and ${{P}_{i}}$ is the fraction of terminal nodes supplied by the *i*th root node. The value of $H$ varies between 0 and 1. $H=1$ means that all vascular trees have the same number of terminal nodes. For $H=0$, all terminal nodes are connected to one vascular tree. Radiation damage was simulated on twenty unconstrained forests, and evaluated as a function of $H$.

#### 2.3.4. Scoring of radiation damage.

Our measure of radiation damage to the unconstrained vascular forests is the mean probability for terminal nodes to be connected to a root node by a chain of intact vessels (mean supply probability). A single terminal node connected to a root node via the blood vessels ${{B}_{1,\ldots,{{n}_{k}}}}$ has a supply probability equal to the product of the survival probabilities of these blood vessels, ${{ \Phi }_{k}}={\prod}_{{{i}_{k}}=1}^{{{n}_{k}}}P\left({{B}_{{{i}_{k}}}}\right)$, with $P\left({{B}_{{{i}_{k}}}}\right)$ being the survival probability of the blood vessel ${{B}_{{{i}_{k}}}}$. The mean supply probability for a vascular forest with t terminal nodes is therefore

4 \begin{eqnarray*}\langle \Phi \rangle ~=\frac{1}{t}\underset{k=1}{\overset{t}{\sum}}\,{{ \Phi }_{k}}=\frac{1}{t}\underset{k=1}{\overset{t}{\sum}}\,\underset{{{i}_{k}}=1}{\overset{{{n}_{k}}}{\prod}}\,P\left({{B}_{{{i}_{k}}}}\right)\end{eqnarray*}

For the simulations on the entire vascular network, we assessed radiation damage to the vasculature by calculating the ratio of the total vascular network length after irradiation to the length of the undamaged vascular network. To analyze the remaining tissue vascularization, distances to the nearest perfused capillary were measured from 27 000 points distributed homogeneously over the simulated tissue volume. All calculations of radiation damage exclude a margin of 250 *µ*m at the boundaries of the simulation volume.

## 3. Results

### 3.1. Anatomical model

For the arterioles and venules, the outer optimization yielded a root mean square deviation of the simulated anatomical parameters from the target values of 1.92% on average. The resulting pressure drops between the root nodes and terminal nodes of the vascular forests were ${{P}_{\text{A}}}=25.73\,\text{mmHg}$ for arterioles and $~{{P}_{\text{V}}}=16.46\,\text{mmHg}$ for venules.

**Figure 6.**
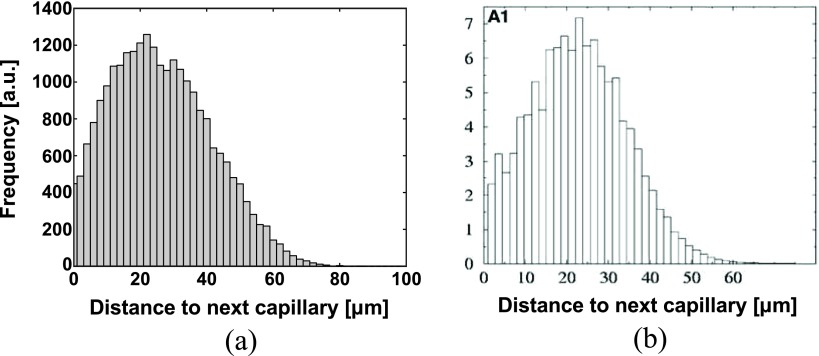
(a) Distribution of distances from points in the tissue to the next capillary resulting from the simulated capillary network. (b) Distribution measured by Risser *et al* (2006), figure 3, section A1.

To validate the capillary model, the distribution of distances from points in the tissue to the nearest capillary was compared to the distribution measured by Risser *et al* (2006) on a human cortical microvascular network. The resulting histograms are presented in figure 6; the estimated relative RMS deviation between the experimental and simulated distribution, after normalization, was 5%. The mean distance from the nearest capillary was $\left(26.81\pm 0.04\right)$ *µ*m in the simulation versus 24 *µ*m in the reference distribution. The ‘cutoff’ of the distance distribution, which we defined as its 98% quantile, was $\left(59.48\pm 0.1\right)$ *µ*m for the simulated distribution and 48.5 *µ*m for the reference. The statistical parameters of the simulated distributions of distances are presented as mean  ±  standard deviation of 10 simulated capillary networks.

### 3.2. Radiation damage model

**Figure 7.**
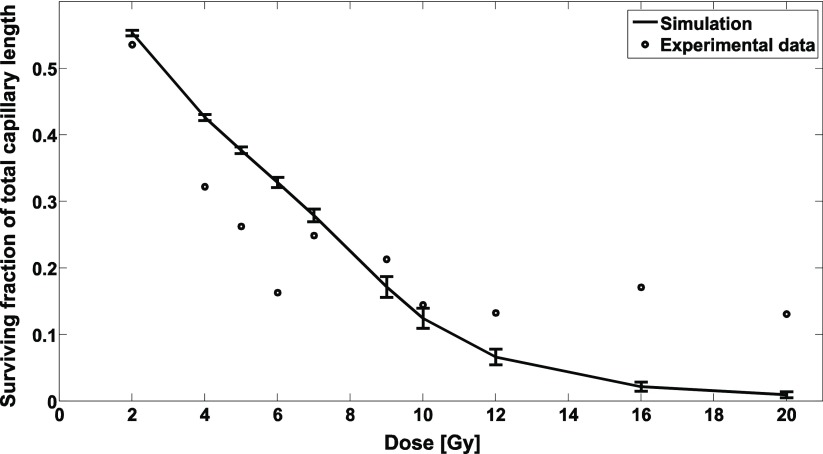
The circles represent experimental data on the length of a capillary network after irradiation relative to the unirradiated network’s length, measured by Dimitrievich *et al* (1984). The curve is the simulated relative capillary network length after BB irradiation with the fitted model parameters. The error bars indicate the standard deviation across five simulated capillary networks.

We calibrated our model parameters *A* and *λ* (see equation (2)) using experimental data on the length of an irradiated capillary network relative to the unirradiated network’s length (Dimitrievich *et al* 1984). The resulting estimates were $A=\left(43.8\pm 1.3\right)~\,\mu \text{m}{}^\text{2}~$ and $\lambda =2.40\pm 0.05$. A comparison of modelling results with experimental data is shown in figure 7.

For doses of 7–10 Gy, the deviation between the simulated and the reference data points is below 3.2*σ*, with *σ* the standard deviation measured across 5 simulated capillary networks. However, the model does not reproduce the nearly constant survival for doses higher of 12 Gy or higher, nor does it reproduce the local minimum at 6 Gy as seen in the experimental data.

### 3.3. Vascular network length after irradiation

**Figure 8.**
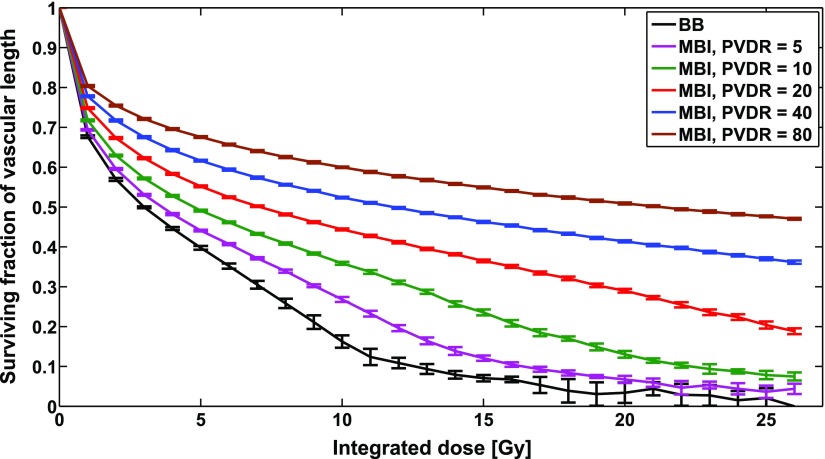
Remaining fraction of total vascular network length versus integrated dose after BB and MBI at different PVDRs. The peak width was 50 *µ*m.

The overall damage to the vasculature due to irradiation was assessed by measuring the ratio of the length of the vascular network after irradiation to its length before irradiation. In figure 8, the remaining fraction of total vascular network length after irradiation is plotted against the integrated dose delivered for MBI with peak-to-valley dose ratios ($\text{PVDR}$) between 1 and 80. The relative vascular network length decreased strongly with decreasing $\text{PVDR}$ when the integrated dose was kept constant and was lowest for BB irradiation, i.e. $\text{PVDR}~=~1$. Numerical simulations with $\text{PVDR}~=~20$ and a peak width of $\text{PPD}/8$ yielded degrees of radiation damage which did not depend on the peak-to-peak distance, nor on the orientation and location of the microbeam array.

### 3.4. Tissue-vessel distances

**Figure 9.**
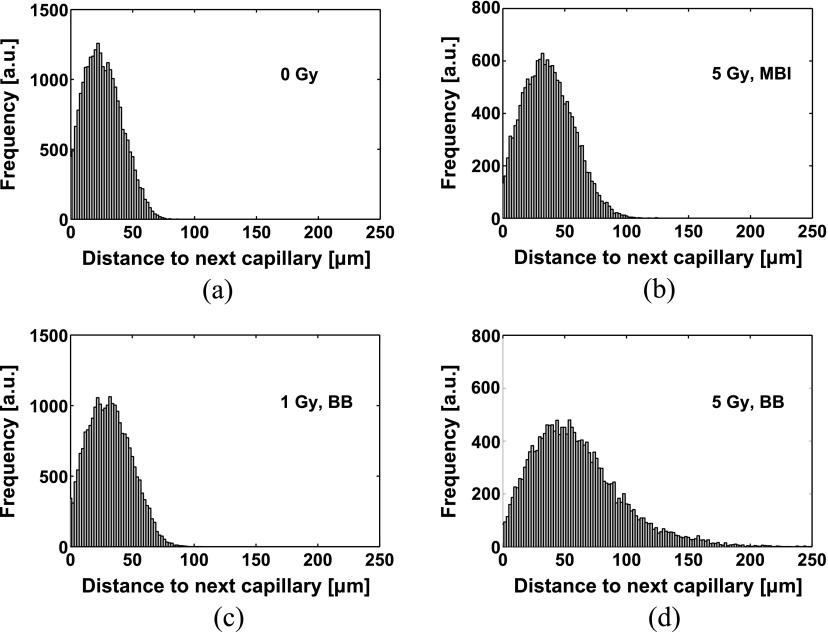
Distributions of distances from points in the tissue to the nearest capillary. (a) No irradiation. (b) Microbeam array, integrated dose 5 Gy. (c) Homogeneous dose, 1 Gy. (d) Homogeneous dose, 5 Gy. The histogram for 5 Gy MBI depicts considerably lower distances, i.e. less damage than the 5 Gy BB histogram.

In figure 9, histograms of distances from homogeneously distributed points in the tissue volume to the nearest perfused capillary are shown for the unirradiated vasculature and the vasculature irradiated with BB and MBI. With increasing dose, the mean value and the width of the distance distribution increased. The effect of a homogeneous dose on the distance distribution was greater than that of the same integrated dose delivered by microbeams. A calculation of equivalent homogeneous doses ${{D}_{\text{H}}}$ for MBI with an integrated dose ${{D}_{\text{I}}}$ with respect to vascular network length and mean tissue-capillary distance yielded a linear relationship ${{D}_{\text{H}}}=a{{D}_{\text{I}}}+b$ for both biological endpoints. The parameters $a$ and $b$ were independent of the biological endpoint: For the vascular network length, $a=0.3526~\pm ~0.0059~$ and $b=\left(0.445~\pm ~0.047\right)\,\text{Gy}$, for the mean tissue-capillary distance, $a=0.3422~\pm ~0.0095$ and $b=\left(~0.469~\pm ~0.085~\right)\,\text{Gy}$.

### 3.5. Characterization of vascular tree morphology

**Figure 10.**
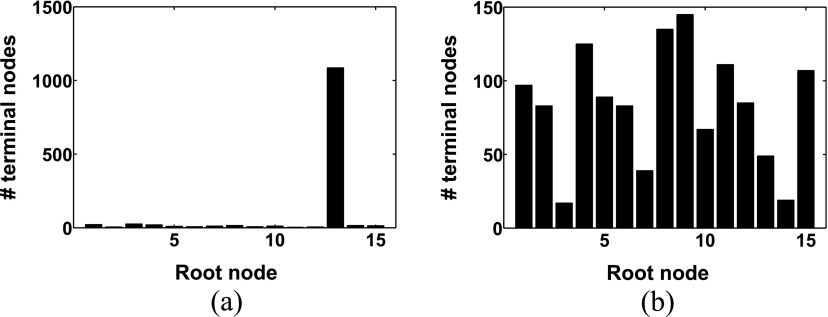
Distributions of venous terminal nodes among the root nodes. (a) Low Shannon entropy $H~=~0.267$. (b) High Shannon entropy $H~=~0.956$.

For 20 unconstrained forests, the number of connected terminal nodes was counted for each venous root node. From these distributions, the Shannon entropy was calculated and used to characterize the vascular forests. Figure 10 shows two of these distributions, one with a very high and one with a very low Shannon entropy.

**Figure 11.**
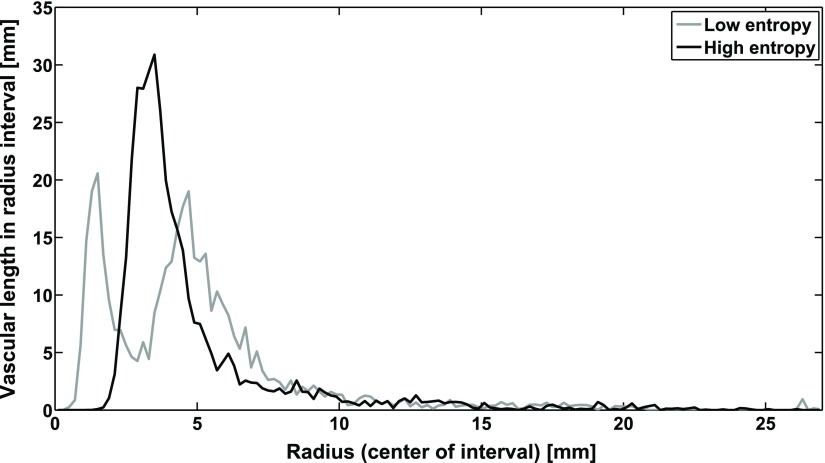
The distribution of radii in the vascular forests for high entropy and low entropy. The total length of arterioles and venules with radii in intervals of 0.2 *µ*m width is plotted against the radius.

The different Shannon entropies were associated with remarkably different distributions of vessel radii along the length of the vascular network as shown in figure 11. The distribution for low entropy was characterized by two peaks, one at 1.6 *µ*m and one at 4.8 *µ*m. The distribution for high entropy had one peak at 3.6 *µ*m. The two peaks for the low-entropy vascular forest, which mainly consisted of one vascular tree, correspond to the blood vessels at the two highest branching levels. In the high-entropy forest, the large number of vascular trees with varying numbers of terminal nodes led to less well-defined vessel radii for the different branching levels.

### 3.6. Radiation damage to vascular forests

**Figure 12.**
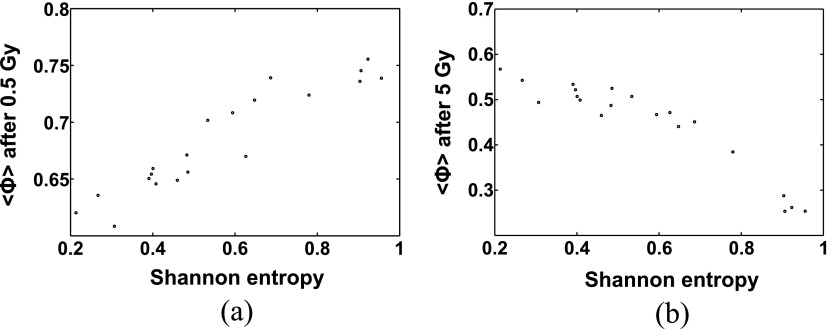
Dependence of the blood supply $\langle \Phi \rangle $ on the Shannon entropy after BB irradiation with (a) 0.5 Gy and (b) 5 Gy.

For a set of 20 unconstrained vascular forests which were irradiated with homogeneous doses, figure 12 shows the blood supply $\langle \Phi \rangle $ (equation (4)) as a function of the Shannon entropy $H$ at 0.5 Gy and 5 Gy. At BB irradiation with 0.5 Gy, asymmetric vascular forests (low entropy) were more prone to radiation damage than balanced forests (high entropy) while at 5 Gy, homogeneous forests of vessels exhibited an enhanced level of radiation damage. Calculations of the correlation coefficient between $H$ and $\langle \Phi \rangle $ as a function of the BB irradiation dose $D$ resulted in a positive correlation for $D&lt;1.05\,\text{Gy}$ and a negative correlation for $D&gt;1.05\,\text{Gy}$.

**Figure 13.**
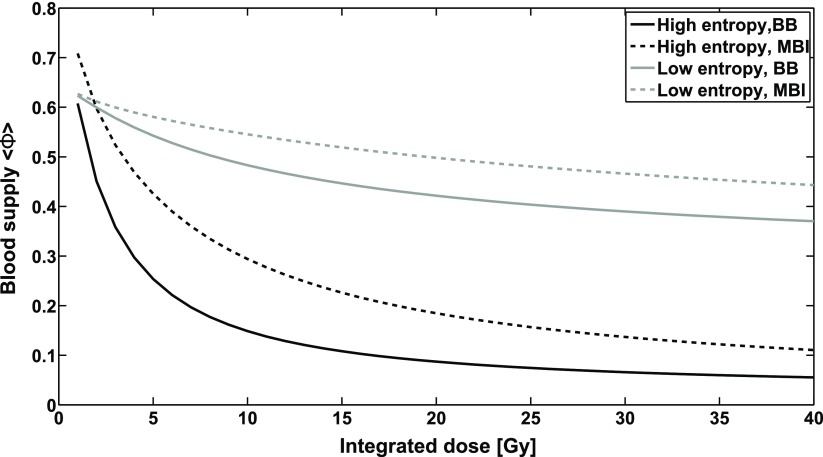
Dose-dependent blood supply in vascular forests with high entropy and low entropy after exposure to MBI and BB irradiation.

In our simulations, homogeneous irradiation caused more damage than MBI with the same integrated dose. Despite the highly anisotropic geometry of the vascular forests, the damage inflicted by MBI did not change with rotations or translations of the microbeam planes. However, the differential effect of MBI depended on both the integrated dose and the Shannon entropy. Figure 13 shows the blood supply $\langle \Phi \rangle $ (equation (4)) for MBI and BB as a function of the integrated dose, for the two vascular forests with high ($H=0.956$) and low ($H=0.267$) entropy (figure 10). For an integrated dose $~{{D}_{\text{I}}}&lt;27\,\text{Gy}$, microbeams spared high-entropy vasculature more than low-entropy vasculature, if BB irradiation with the dose ${{D}_{\text{I}}}$ is used as a reference point. At higher doses, the differential effect was more pronounced for the low entropy vasculature, i.e. ${{\langle \Phi \rangle}_{\text{MBI}}}-{{\langle \Phi \rangle}_{\text{BB}}}$ was greater.

## 4. Discussion

Two models have been developed in this work, a model of the vascular anatomy of the cerebral cortex and a model that predicts the survival probability of a blood vessel section after being irradiated by ionizing radiation. These two models were used to simulate the radiation damage inflicted on the vasculature of the cerebral cortex by a microbeam field (MBI) and a homogeneous radiation field.

The model for the vasculature of the cerebral cortex yields a simulated morphology which closely resembles observed *in vivo* vascular network morphologies. Selected anatomical parameters of the simulated vascular trees, the radii of root nodes and terminal nodes and the density of arterial and venous branching nodes deviate on average by less than 1.92% from empirically measured parameters. Comparing the flow resistance of the simulated vasculature with previously published data reveals close agreement. The simulated arterial and venous pressure drops in the model are 25.73 mmHg and 16.46 mmHg, while Zagzoule and Vergnes (1986) calculated 30 and 15 mmHg. Apart from that, experimentally determined distributions of distances of points in the tissue to the respective nearest capillaries (Risser *et al* 2006) were compared to simulated distributions. The visual appearance of the histograms in figure 6 suggests that the simulated distribution is similar to the experimental one. A normalized root-mean-square deviation of 5% additionally indicates good agreement. The differences between statistical parameters which were calculated on the experimental and simulated distributions were less than 20% relative to the simulated values, though statistically significant compared to the variability of the simulated capillary geometry caused by the random part of the simulation algorithm. Since experimental data on the inter-subject variability of these parameters were not available, the values presented in the results section necessarily overestimate the significance of these differences. Furthermore, differences between the primate species and the exact anatomical location which yielded the reference data by Risser *et al* (2006) and the data on the capillary network length used for our simulation (Weber *et al* 2008) are an additional substantial source of uncertainty. If our slight overestimation of the distances between parenchymal cells and capillaries is not within these unknown biological uncertainties, it may be tentatively explained by the inclusion of some arterioles and venules in the experimental reference data. Our algorithm using Voronoi diagrams thus seems to be a useful approach for simulating the geometry of capillary networks, which simulates an approximately correct and highly reproducible degree of tissue vascularization, while the exact location of the capillaries is random.

The response of a microvascular system to irradiation is a complex combination of both lethal and sub-lethal morphological alterations which can impair the function of blood vessels. Endothelial cells have been shown to be the most radiosensitive components of the blood vessel wall (Fajardo 2004). Short-term effects are a transient change in the permeability of the vessel wall (e.g. Brinkman and Lamberts (1966), Baker and Krochak (1989) and Bouchet *et al* (2015)), irregular EC proliferation which can lead to protrusions into the vessel lumen or irregularly shaped blood vessels (Baker and Krochak 1989), as well as apoptosis (Langley *et al* 1997). The removal of endothelium from the vessel wall can lead to an occluding thrombus (O’Connor and Mayberg 2000), or, in capillaries, a rupture of the vessel wall (Dimitrievich *et al* 1984) less than 1 week post-irradiation. Repair of a damaged area of endothelium occurs on a time scale of days, by proliferation, migration, and stretching of endothelial cells (Reinhold and Buisman 1975, Reidy and Schwartz 1981, Itoh *et al* 2010). In addition, endothelial cells exhibit delayed damage several months post-irradiation (Münter *et al* 1999, Nguyen *et al* 2000). Relevant reviews on the effects of irradiation on the vasculature include the works by Hopewell (1983), Baker and Krochak (1989), O’Connor and Mayberg (2000) and Fajardo (2004).

The radiation damage model presented in this publication is based on the assumption that vascular damage is largely an effect of damage to the endothelium. In this model, the destroyed area of endothelium determines whether a blood vessel will remain intact or become dysfunctional. The model is therefore independent of the specific biological processes involved. Two possible biological interpretations are an occluding thrombus or a leak in the vessel wall. Since the purpose of the model is to simulate the influence of the vascular network geometry and the dose distribution on radiation damage, it naturally uses major simplifying assumptions regarding the properties of blood vessels and their response to irradiation. Most importantly, it is a strictly spatial model and does not include time-dependent effects (endothelial cell repair, vascular remodelling) or a time evolution of vascular damage. Another simplification concerns the rigid cylindrical geometry of the modelled blood vessels, which neglects functional changes in calibre, pulsations, as well as the shapes of real blood vessels (Duvernoy *et al* 1981), but is commonly used for simulating vascular anatomies (e.g. Zagzoule and Vergnes (1986), Kretowski *et al* (2003) and Hamarneh and Jassi (2010)), as it allows an analytical description of the relationship between blood flow and pressure gradients. Similarly, the modelling of the vascular radiation response as a two-state system (intact/destroyed) for both the endothelial cells and the entire blood vessel segments is a significant deviation from the biology of a small blood vessel, but a useful assumption for modelling the behaviour of an entire vascular network.

The radiation damage model predicts the probability per length interval that a blood vessel remains intact as a function of its cross section. The model further depends on the two physiological model parameters *A*, *λ* and the endothelial cell survival $\nu (D)$. Due to the lack of existing quantitative data on radiation damage to the vasculature in the human cerebral cortex, we used data by Dimitrievich *et al* (1984) on microvessels in the rabbit ear chamber after homogeneous irradiation to calibrate the developed model and fit the parameters *A* and *λ*. In contrast, $\nu (D)$ describes clonogenic cell survival data derived from endothelial cell survival in the irradiated mouse brain (Lyubimova *et al* 2001). This discrepancy in animal models is a major source of uncertainty for the estimation of the model parameters which should be overcome in further experimental studies. In addition, the reference data on the radiation response of blood vessels require closer consideration. For the local minimum of the experimental relative capillary network length after irradiation with 6 Gy as well as the survival tail for 12–20 Gy, Dimitrievich *et al* (1984) provide no biological explanation. It remains uncertain if these features are reproducible, and if they were, which biological effects have caused them. Hence, while our purely geometric model, with its monotonously decreasing survival curves, is not designed to reproduce the experimental survival curves in detail, it remains an open question if developing a model which achieves this would be meaningful from a biological point of view. Our fit therefore only serves the purpose of calibrating the model to an experimentally observed scale of radiation doses. With this preliminary estimate of model parameters, we were able to determine geometrical parameters which may influence radiation damage, but precise quantifications of radiobiological effects are not yet possible. More detailed experimental and physiological data on the geometric properties of vascular networks after irradiation will be required, in a defined species, to achieve a detailed experimental validation and to improve the quantitative predictions of the developed model.

The radiation damage of the vascular system was examined by evaluating the total vessel length reduction of the vasculature and recording changes in the distribution of tissue-vessel distances. Oxygen diffuses from the capillaries to the parenchymal tissue up to a well-defined radius between 50 and 300 *µ*m (Tannock 1972, Kasischke *et al* 2001). Therefore, an increase in the tissue-vessel distances may lead to hypoxia. Simulation results show consistently that a certain length reduction is associated with similar tissue-vessel distance distributions, independent of MBI or homogenous irradiation and therefore both end-points can be used interchangeably.

When comparing microbeam and broad beam irradiation the developed model proves capable of predicting several experimental findings.

(1) MBI inflicts less radiation damage on the vascular system than homogeneous beams and higher PVDRs spare the vasculature more effectively. In order to cause equivalent damage MBI requires a 2.9 times higher integrated dose than homogeneous irradiations. However, doses in the simulation are much lower than those observed *in vivo*. Serduc *et al* (2006) observed for example that microbeams with 1000 Gy peak dose are tolerated by the microvasculature of the normal mouse brain without signs of impairment for at least 1 month after irradiation. In their experiment they used a beam width of 25 *µ*m, a beam to beam spacing of 211 *µ*m and an integrated dose of 118.5 Gy, while an integrated dose of around 10 Gy in the simulation is already able to reduce the vascular length by 50%. Several reasons may be responsible for these discrepancies. The model in this work is based on physiological data of different organisms and therefore a direct quantitative comparison is of limited meaning. Furthermore, the radiation damage model uses clonogenic data on the survival of endothelial cells, which generally predict lower radiation tolerances than *in vivo* observations. Repair mechanisms that were completely neglected in the present model may also lead to a higher radiation tolerance *in vivo*.
(2) The radiation sensitivity of the vascular system depends on its symmetry as measured by Shannon entropy. Since the geometry of tumorous vasculature differs greatly from that of healthy vasculature (Risser *et al* 2006), this finding shows that there may be a geometrical component to the selective effect of MRT on tumours. Whether this is indeed the case, and if this effect is significant compared to physiological factors, in particular vascular maturation (Sabatasso *et al* 2011, Brönnimann *et al* 2016) should be further investigated.
(3) Orientation and location of the microbeams do not influence the overall tumour survival and the level of damage does not depend on the peak width as long as the ratio between peak to peak spacing and peak width remains constant. Serduc *et al* (2014) examined the *in vivo* effect of microbeams on the rat brain at peak widths in a range between 25–1000 *µ*m and a peak spacing between 400 and 2000 *µ*m. They concluded that thinner microbeams cause less damage than thicker microbeams. However, the PVDRs used in their study varied between 132 at 25 *µ*m and 40 at 1000 *µ*m peak width and therefore their observations do not contradict the prediction that the damage is independent of the peak width. Nevertheless, migration and repair of endothelial cells which is currently not part of the developed model will probably be more effective for thin microbeams.

As one reason for the sparing of the vasculature after microbeam irradiation we identify the convexity of the dose response relationship, i.e. $\frac{{{\text{d}}^{2}}S}{\text{d}{{D}^{2}}}&gt;0$. The probability P that a vessel remains intact depends on the dose, $P~=~P(D)$ as shown in figure 4. The overall integrity of the vascular system can be estimated by averaging over the probability $P$ of all vessels in the considered volume. The average survival probability in an inhomogeneous, spatially modulated radiation field will be higher than in a homogeneous radiation field since a convex dose response yields $\overline{P(D)}\geqslant P\left(\bar{D}\right)$, where the bar indicates averaging over all vessels in the considered volume.

The influence of the vascular forest morphology on the radiation damage can be explained by differences in the distribution of blood vessel radii (figure 11). At low doses only the thinnest, most radiosensitive blood vessels are destroyed. Since the asymmetric vascular forests with low Shannon entropy have a higher fraction of thin blood vessels they are more sensitive to radiation. For higher doses, capable to effectively destroy vessels with a radius of 3 *µ*m, this behaviour changes and the damage to high entropy vascular forests will be greater than to low entropy vascular forests.

The predictive value of the developed model could be enhanced by using more comprehensive data on clonogenic survival of endothelial cells, in particular for radiation doses above 10 Gy. Possible further improvements concern the inclusion of damage repair mechanisms and post-irradiative vascular growth. This would require a time-dependent model as well as further experimental studies on vascular network properties as a function of radiation dose and its dynamics after radiation treatment.

## 5. Conclusions

In this work numerical modelling has been used for the first time to investigate the lower vascular damage observed after microbeam radiation therapy as compared to homogeneous irradiation at equal integrated dose levels and the differential effect of microbeams on tumour and healthy tissue vasculature. The model shows that a 2.9 times higher integrated microbeam dose at a PVDR of 20 is required to cause the same damage level as a homogeneous irradiation. The study of different vascular tree morphologies showed that the distribution of blood vessel radii essentially determines the overall radiosensitivity of a vascular forest and the specific response to MBI and homogeneous irradiation. This may partly explain the differential effect MRT has on tumours and healthy tissue.

The assumption made in the developed models are not specific to MRT and therefore other applications are feasible, such as studying the effect of vascular damage on tumour oxygenation or a modelling of drug delivery by the use of radiotherapy induced permeability between blood and brain, and blood and tumour.

Further experimental studies on the geometric properties of vascular networks after broad beam and microbeam irradiation are necessary to improve the quantitative predictions of the model.
